# Coverage and determinants of hepatitis B virus vaccine uptake among children aged 12–36 months in Ethiopia: an analysis of the 2016 Ethiopian Demographic and Health Survey

**DOI:** 10.1093/inthealth/ihaf142

**Published:** 2025-12-13

**Authors:** Kathleen Kenyatta White, Tesfa Sewunet Alamneh, Josephine G Walker, Aaron G Lim

**Affiliations:** Population Health Sciences, Bristol Medical School, University of Bristol, Canynge Hall, Bristol BS8 2PS, UK; Population Health Sciences, Bristol Medical School, University of Bristol, Canynge Hall, Bristol BS8 2PS, UK; Department of Epidemiology and Biostatistics, Institute of Public Health, College of Medicine and Health Sciences, University of Gondar, Gondar, Ethiopia; Population Health Sciences, Bristol Medical School, University of Bristol, Canynge Hall, Bristol BS8 2PS, UK; Population Health Sciences, Bristol Medical School, University of Bristol, Canynge Hall, Bristol BS8 2PS, UK

**Keywords:** DHS, Ethiopia, HBV, public health, spatial distribution, sub-Saharan Africa, vaccination

## Abstract

**Background:**

In Ethiopia, chronic hepatitis B virus (HBV) prevalence is high (∼6%) and infant vaccination targets are unmet. We assessed HBV vaccination uptake and associations with parental sociodemographic and obstetric factors among children (aged 12–36 months) in Ethiopia.

**Methods:**

We utilised the 2016 Ethiopian Demographic and Health Survey. We included a weighted sample of 3,855 children (aged 12–36 month) to assess HBV vaccination coverage level (none, incomplete, and complete) and its spatial distribution. Additionally, we identified associated factors of HBV vaccination coverage level using ordinal logistic regression.

**Results:**

Overall, 51.2% of children received complete HBV vaccination, with wide regional variation. Older maternal age (adjusted odds ratio (AOR)=1.05, 95% confidence interval (CI): 1.02–1.07), better maternal education level (mean AOR=1.66–2.08), higher household wealth quantile (mean AOR=1.51–2.32), distance to a healthcare facility not a big problem (AOR=1.54, 95% CI: 1.25–1.89), and complete maternal continuum care utilisation (AOR=2.59, 95% CI: 1.25–5.30) were positively associated with HBV vaccination coverage, while higher number of children in the household (AOR=0.86, 95% CI: 0.79–0.92) was negatively associated.

**Conclusions:**

HBV vaccination coverage falls below World Health Organization (WHO) targets in Ethiopia and has substantial regional variation. Targeted vaccination campaigns for mothers in lower socioeconomic groups and larger families may improve vaccination coverage.

## Introduction

Chronic hepatitis B virus (HBV) infection is responsible for significant global morbidity and mortality, with an estimated 254 million people living with chronic HBV infection in 2022.^1^ End-stage liver disease complications associated with chronic HBV infection, including decompensated cirrhosis and hepatocellular carcinoma, cause 1.1 million deaths annually.^1–3^ Globally, HBV prevalence is geographically heterogeneous, with the most prevalent chronic HBV infections located in the WHO Western-Pacific, African and Southeast Asian regions.^1^ According to World Bank estimates, Ethiopia is the second most populous country in Africa, and is believed to have a high burden of HBV. A systematic review and meta-analysis of studies published from 2010 to 2019 showed an overall pooled HBV prevalence of 6% across 60 studies, ranging from 1 to 36% across regions of Ethiopia.^3^ As a large and populous lower- and middle-income country, Ethiopia faces considerable challenges in access to healthcare and delivery of services related to chronic HBV infection.^4,5^

HBV is highly infectious and is transmitted by contact with infected blood or seminal fluids; infection can be vertical from mothers to neonates via the placenta and amniotic fluid, or horizontal from person-to-person.^6^ In areas of high endemicity, defined by the WHO as having >8% HBV prevalence, the primary route of transmission is vertically, from mother to child perinatally, or in early postpartum, and infants can acquire HBV through close contact with infected family members.^3,6,7^ Development of chronic HBV infection is inversely associated with age at infection, with 95% of neonates, 20–30% of children and 5% of adults developing chronic infection.^1,6^ There is no cure for chronic HBV and management of chronic cases is costly, involving long-term treatment, assessment of active infection (HBV replication status), screening for HIV and other coinfections, as well as continuous assessment of liver function and liver disease.^6^

The WHO Global Health Sector Strategy aims to reduce HBV prevalence in 0–4-y-olds to 0.1% by 2030 and recommends complete infant vaccination against HBV to achieve this target.^8,9^ The HBV vaccine is delivered in a three-dose programme in the first 6 mo to confer complete protection for infants.^10,11^ However, dosing schedules for HBV vaccination varies globally, with recommendations for dosing in Ethiopia occurring at birth, 6 and 10 wk, with an optional fourth dose at 14 wk.^12^ Prevention through effective vaccinations (administering birth dose for HBV-exposed infants to reduce the risk of mother-to-child transmission and triple doses of HBV vaccine) can reduce the long-term costs associated with chronic case management and treatment, which is essential for reducing chronic HBV-associated disease progression.^10^

Following WHO recommendations, the Ethiopian government has made reducing the burden caused by HBV a public health priority, and the inclusion of the HBV vaccine series to the Expanded Programme for Immunisation (EPI) was implemented in 2007.^2,13^ Since implementation, the WHO has reported on infant HBV vaccination coverage; however, this has remained low, reaching a reported 64% in 2016.^14^ Further to this, evidence from the 2011 Ethiopian Demographic and Health Survey (EDHS) indicated that complete vaccination among infants was 24%, with wide variability between rural (20%) vs urban (48%) coverage. This demonstrates that infant vaccination coverage has not been at an adequate level to prevent the spread of HBV infection and indicates there is a requirement for an improved understanding of the factors affecting vaccine uptake.^13,15^

Multiple factors have been shown to influence vaccine uptake in children aged <5 y across the African continent, including maternal and paternal education, socioeconomic status, access to health facilities and ante- and post-natal care.^13^ This analysis aimed to identify the underlying factors affecting HBV vaccine uptake in infants (aged 12–36 mo) in Ethiopia using a nationally representative dataset. This study aims to provide an evidence base for the underlying factors affecting vaccine uptake, which could be used to inform policymakers and to potentially implement change in vaccination campaigns.

## Methods and materials

### Data source and study population

This study used 2016 EDHS data.^16^ Demographic and Health Surveys are nationally representative household surveys that are conducted in >90 countries worldwide, providing data on population, health and nutrition for impact monitoring and evaluation. The EDHS is conducted by the Central Statistics Agency of Ethiopia every 5 y, with the 2016 issue being the fourth round of survey in Ethiopia, and the most recently available dataset.^16^

The target population for the EDHS consisted of women aged 15–49 y, men aged 15–59 y and any child aged <5 y living in the selected households. If there was >1 child aged <5 y, data were collected on the youngest child. Five questionnaires were used for the EDHS: Household, Women, Men, Biomarker and Health Facility, to collect detailed sociodemographic, domestic and health information.^16^ In this analysis, we used the Children’s Recode file, which contained data on the child and their mother. Paternal sociodemographic and obstetric factors were also extracted from this dataset for live children aged 12–36 mo.

### Study variables

The outcome variable was HBV vaccination status, which combined data on the number of vaccine doses that each child received. This was coded into ‘No Vaccination’ for children who had zero doses of the HBV vaccine, ‘Incomplete Vaccination’ for children who had received one to two doses and ‘Complete Vaccination’ for children who received three doses (see Supplementary Methods). The response variable was split into three categories as there is evidence to indicate that having ‘incomplete vaccination’ against HBV, as compared with having no vaccination, offers some protective effects.^11^

Exposure variables utilised in this study were parental sociodemographic and obstetric factors. The sociodemographic variables included maternal age at birth of the child; maternal education; paternal education; wealth index; marital status; exposure to mass media; and number of living children in the household. The obstetric variables were distance to a healthcare facility and maternal continuum of care utilisation, which combined antenatal care visits, delivery of a child in a healthcare facility and postnatal care visits. Further details regarding the study variables can be found in the Supplementary Materials.

### Data management and statistical analysis

Data access was requested from the DHS Program (https://dhsprogram.com/) and with permission, all variables required for this study were extracted from the Children’s Recode file of the EDHS 2016 dataset. Data were weighted using the EDHS sample weighting variable to adjust for unequal probability of selection under the study design.^4^

Univariable and multivariable ordinal logistic (cumulative logit) regression were undertaken to test the association between parental sociodemographic and obstetric factors and HBV vaccination level. Ordinal logistic regression was selected due to the ordered nature of the categories of the outcome variable. The proportional odds assumption of the cumulative logit model was tested using the Brant test and found to be satisfied.^17^ Variables with p<0.2 in the univariable models were included in the multivariable model. The statistical analysis was performed in STATA version 17.0 (StataCorp LLC, College Station, Texas, USA).^18^ A sensitivity analysis was performed, removing paternal education from the model due to small amounts of missingness in the data for this variable.

### Spatial analysis

Geographic coordinates (both longitude and latitude) data collected at the enumeration area level from the 2016 EHS were utilised for spatial analysis. We evaluated spatial autocorrelation (i.e. whether the distribution was random or not) of complete HBV vaccination uptake among children in Ethiopia using the Global Moran’s I statistic. Following this, hotspot analysis was conducted using Getis-Ord Gi* statistics to locate areas with higher (significant hotspot areas) and lower (cold spot areas) rates of no or incomplete HBV vaccination coverage.^19^ The spatial analyses were conducted using ArcGIS V.10.7 software.^20^

### Ethical considerations

This study is an analysis of the 2016 EDHS, for which ethical approval was previously granted for data collection. To access and download data, online registration was completed and data were downloaded from the EDHS online archive once approvals were obtained.

## Results

### Descriptive characteristics of the study population

A total of 3855 children (EDHS-weighted) aged 12–36 mo with available HBV vaccination dose data were included. Sociodemographic characteristics showed that 2379 (61.7%) mothers and 1730 (47.7%) fathers had no formal education. Meanwhile, 3628 (94.1%) mothers were currently married or living with a partner and 3038 (78.8%) did not access mass media regularly (Supplementary Table S1). Obstetric factors showed that 3706 (96.1%) mothers did not access the full maternal continuum of care and 2075 (53.8%) mothers had a problem with the distance to a healthcare facility (Supplementary Table S1).

### Magnitude of HBV vaccine uptake

The magnitude of HBV vaccine uptake showed that 2663 (69.1%) children received at least one dose, 2325 (60.3%) received at least two doses and 1972 (51.2%) received all three doses of the HBV vaccine. This resulted in 1192 (30.9%) children having had no HBV vaccination, 691 (17.9%) children with incomplete HBV vaccination and 1972 (51.2%) children with complete HBV vaccination (Table 1).

**Table 1.** Factors associated with the hepatitis B virus vaccination status of children (aged 12–36 mo) in Ethiopia

		Hepatitis B vaccine status					
		No vaccination		Incomplete vaccination		Complete vaccination	
Variable	Category (N)	Frequency	Proportion (%)	Frequency	Proportion (%)	Frequency	Proportion (%)
Sex of child	Male (1957)	591	30.2	365	18.65	1001	51.15
	Female (1898)	601	31.7	326	17.18	971	51.16
Maternal age at birth of child (y)	12–15 (33)	9	27.27	10	30.30	14	42.42
	16–20 (685)	241	35.18	130	18.98	314	45.84
	21–25 (1077)	292	27.11	203	18.85	582	54.04
	26–30 (1009)	319	31.62	162	16.06	528	52.33
	31–35 (615)	189	30.73	108	17.56	318	51.71
	36–40 (336)	110	32.74	60	17.86	166	49.40
	41–45 (91)	27	29.67	18	19.78	46	50.55
	46–50 (9)	5	55.56	0	0.00	4	44.44
Maternal education	No education (2379)	947	39.81	472	19.84	960	40.35
	Primary (1019)	189	18.55	181	17.76	649	63.69
	Secondary (294)	38	12.93	28	9.52	228	77.55
	Higher (163)	18	11.04	10	6.13	135	82.82
Paternal education	No education (1730)	723	41.79	355	20.52	652	37.69
	Primary (2379)	263	22.42	201	17.14	709	60.44
	Secondary (399)	75	18.80	55	13.78	269	67.42
	Higher (300)	58	19.33	32	10.67	210	70.00
	Don’t know (26)	8	30.77	2	7.69	16	61.54
Wealth index	Poorest (1435)	721	50.24	305	21.25	409	28.50
	Poorer (640)	173	27.03	115	17.97	352	55.00
	Middle (524)	127	24.24	93	17.75	304	58.02
	Richer (463)	94	20.30	90	19.44	279	60.26
	Richest (793)	77	9.71	88	11.10	628	79.19
Marital status	Never in union (29)	4	13.79	3	10.34	22	75.86
	Married/living together (3628)	1127	31.06	645	17.78	1856	51.16
	Previously married/separated (198)	61	30.81	43	21.72	94	47.47
Mass media exposure	No (3038)	1084	35.68	583	19.19	1371	45.13
	Yes (817)	108	13.22	108	13.22	601	73.56
Distance to health facility	Not a big problem (1780)	394	22.13	297	16.69	1089	61.18
	Big problem (2075)	798	38.46	394	18.99	883	42.55
Maternal continuum of care	No (3706)	1184	31.95	674	18.19	1848	49.87
	Yes (149)	8	5.37	17	11.41	124	83.22
Number of living children	1 (746)	183	24.53	117	15.68	446	59.79
	2 (764)	187	24.48	125	16.36	452	59.16
	3 (590)	182	30.85	97	16.44	311	52.71
	4 (528)	174	32.95	94	17.80	260	49.24
	5 (415)	137	33.01	91	21.93	187	45.06
	6 (324)	132	40.74	70	21.60	122	37.65
	7 (241)	102	42.32	45	18.67	94	39.00
	8 (132)	46	34.85	29	21.97	57	43.18
	9 (74)	27	36.49	19	25.68	28	37.84
	10 (28)	16	57.14	3	10.71	9	32.14
	11 (11)	5	45.45	1	9.09	5	45.45
	12 (2)	1	50.00	0	0.00	1	50.00
All	(3855)	1192	30.92	691	17.92	1972	51.15

### Determinants of HBV vaccine uptake

Our multivariable analyses results indicated that, for children aged 12–36 mo, there was a 5% (adjusted OR [AOR]=1.05, 95% CI 1.02 to 1.07) increase in the odds of being in a higher HBV vaccination level for a 1-y increase in maternal age at birth.

We found that children whose mothers received primary and secondary education, respectively, had 66% (AOR=1.66, 95% CI 1.29 to 2.14) and 108% (AOR=2.08, 95% CI 1.15 to 3.78) higher odds of complete HBV vaccination compared with children whose mothers had no formal education. Children whose fathers received primary education had 43% (AOR=1.43, 95% CI 1.14 to 1.80) higher odds of complete HBV vaccination compared with children whose fathers had no formal education (Table 2).

**Table 2.** COR and AOR for ordinal logistic regression testing the association between sociodemographic and obstetric factors and the HBVs vaccination status of children (aged 12–36 mo) in Ethiopia

Variable	Category	COR (95% CI)	AOR (95% CI)	p (AOR)
Maternal age		0.99 (0.98, 1.01)	1.05 (1.02, 1.07)	<0.001
Maternal education	No education	1	1	
	Primary	2.13 (1.73, 2.62)	1.66 (1.29, 2.14)	<0.001
	Secondary	6.02 (3.51, 10.34)	2.08 (1.15, 3.78)	0.014
	Higher	7.61 (3.37, 17.16)	2.29 (0.92, 5.74)	0.077
Paternal education	No education	1	1	
	Primary	1.81 (1.48, 2.22)	1.43 (1.14, 1.80)	0.002
	Secondary	3.24 (2.22, 4.72)	1.30 (0.83, 2.05)	0.266
	Higher	6.27 (3.63, 10.81)	1.52 (0.79, 2.95)	0.208
Wealth index	Poorest	1	1	
	Poorer	1.72 (1.32, 2.24)	1.55 (1.17, 2.04)	0.001
	Middle	1.80 (1.38, 2.34)	1.51 (1.13, 2.02)	0.004
	Richer	2.48 (1.89, 3.25)	1.73 (1.28, 2.33)	<0.001
	Richest	5.90 (4.23, 8.24)	2.32 (1.51, 3.56)	<0.001
Marital status	Never in union	1	1	
	Married/living with partner	0.78 (0.26, 2.37)	omitted	
	Previously married	0.51 (0.16, 1.64)	omitted	
Mass media exposure	No	1	1	
	Yes	2.40 (1.87, 3.09)	1.18 (0.85, 1.64)	0.325
Distance to health facility	Big problem (further)	1	1	
	Not a big problem (closer)	2.20 (1.82, 2.64)	1.54 (1.25, 1.89)	<0.001
Maternal continuum of care	No	1	1	
	Yes	6.06 (3.23, 11.39)	2.59 (1.27, 5.30)	0.010
Number of living children		0.88 (0.84, 0.91)	0.86 (0.79, 0.92)	<0.001
Intercept 1			0.72 (0.20, 1.25)	
Intercept 2			1.72 (1.19, 2.24)	

AOR: adjusted OR; COR: crude OR; HBV: hepatitis B virus.

Each incremental wealth index category was associated with increased odds of HBV vaccination. Compared with children in the poorest category of wealth index, there were increased odds of complete HBV vaccination among children in the poorer (AOR=1.55, 95% CI 1.17 to 1.80), middle (AOR=1.51, 95% CI 1.13 to 2.02), richer (AOR=1.73, 95% CI 1.28 to 2.33) and richest (AOR=2.33, 95% CI 1.51 to 3.56) categories (Table 2).

For children whose mothers did not consider the distance to a healthcare facility a big problem there were 54% (AOR=1.54, 95% CI 1.25 to 1.89) increased odds of complete HBV vaccination compared with children whose mothers considered it a big problem (Table 2). The largest increased odds were seen for children whose mothers accessed the maternal continuum of care, with 159% (AOR=2.59, 95% CI 1.27 to 5.30) increased odds of complete HBV vaccination compared with children whose mothers did not access it (Table 2).

Conversely, an increase in the number of living children was associated with decreased odds of being in a higher HBV vaccination category. For each additional child, there was a 14% (AOR=0.86, 95% CI 0.79 to 0.92) decrease in the odds of having complete HBV vaccination for their child (Table 2).

### Spatial distribution of complete HBV vaccine uptake

**Figure 1.**
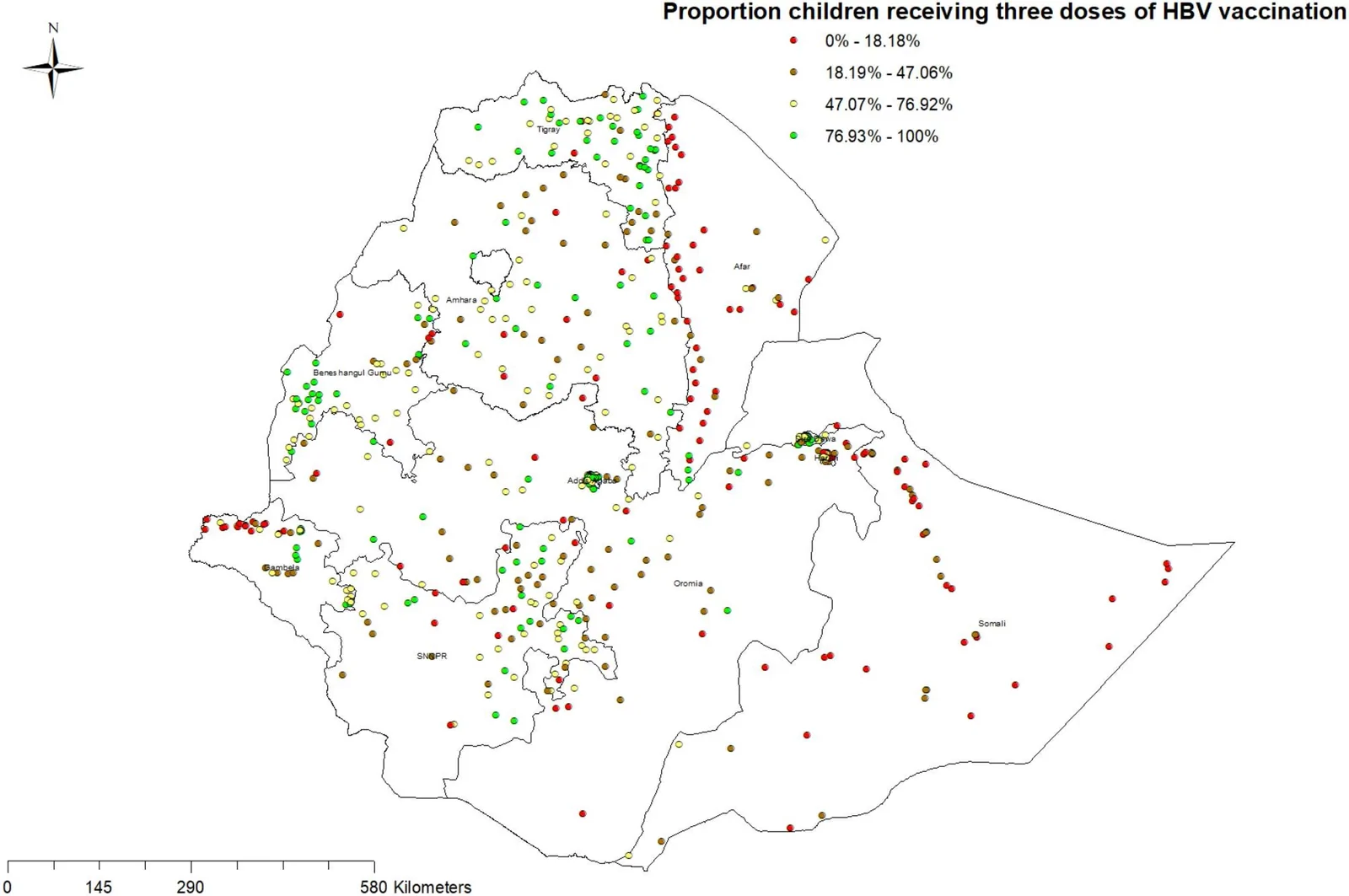
Spatial distribution of complete HBV vaccination among children in Ethiopia, produced using Arc GIS version 10.7. HBV: hepatitis B virus.

**Figure 2.**
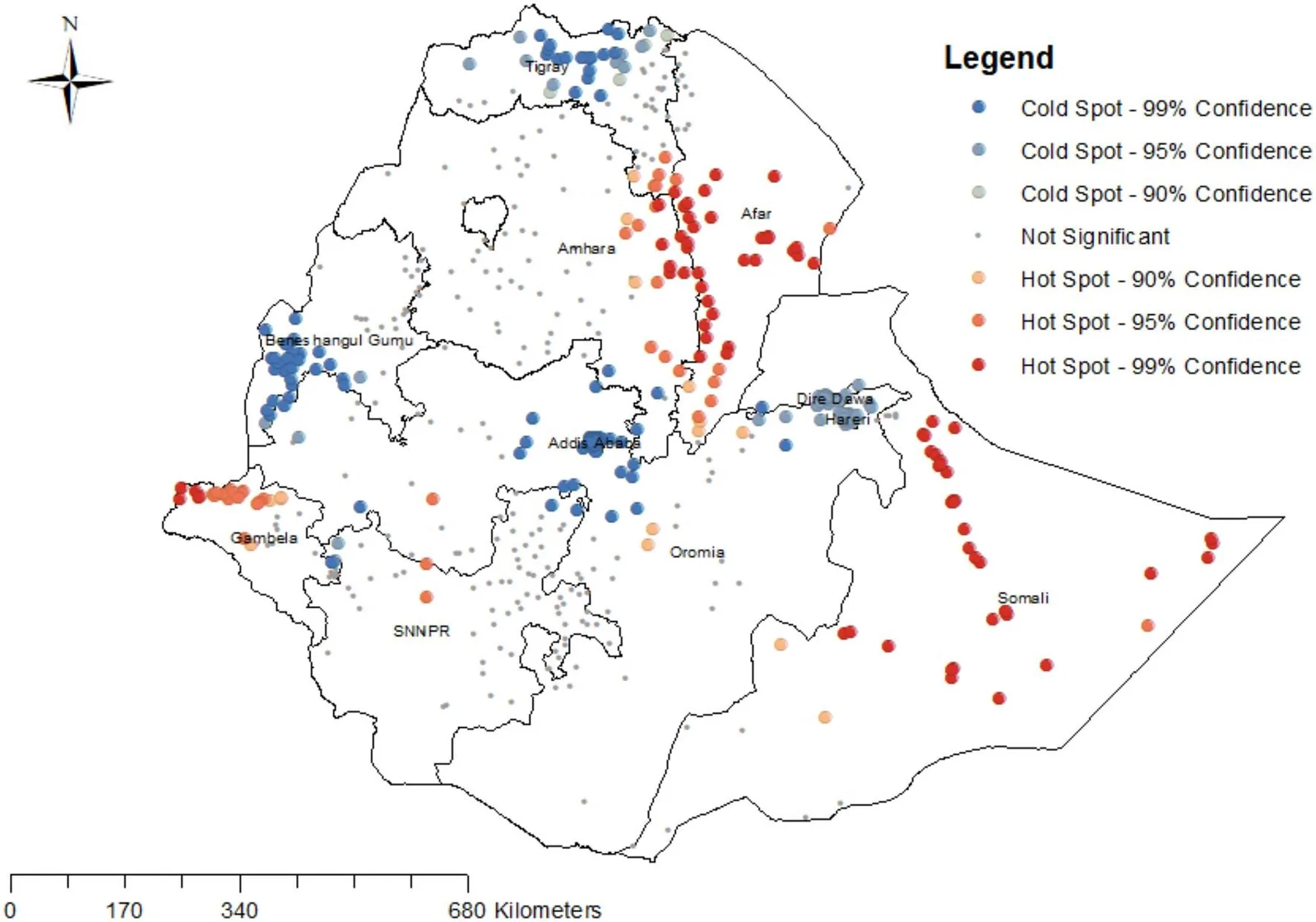
Hotspot analysis of children having no or incomplete HBV vaccination using Getis Ord Gi* statistics in Ethiopia, produced using Arc GIS version 10.7. HBV: hepatitis B virus.

The spatial analysis revealed that receiving complete HBV vaccination was unevenly distributed across the country (Global Moran’s I=0.51, p<0.001) (Supplementary Figure S1). A lower proportion of children completed HBV vaccination in the Afar, Somali and Gambelia regions of Ethiopia, while higher rates of complete vaccination were observed in the Benishangul Gumz, Dire Daw and Addis Ababa regions (Figure 1). Figure 2 illustrates the hotspot and cold spot areas. Enumeration areas with higher rates of no or incomplete HBV vaccination were defined as hotspots, while those areas with lower rates were considered as cold spots. Hotspot areas with no or incomplete HBV vaccination were identified in most clusters from the Afar, Somali and Gambelia regions and the southern part of the Oromia region (Figure 2).

## Discussion

This analysis showed that full HBV vaccination series coverage in Ethiopia remained low (51.2%) in 2016, despite WHO recommendations, the EPI and the specific focus of the Ethiopian government on alleviating the HBV disease burden. These findings show that complete vaccine coverage in Ethiopia falls below commonly reported estimates, including coverage reported by the WHO and estimates from the Global Burden of Disease study.^14,21^ Our spatial analysis suggested that its coverage varies hugely across the regions of Ethiopia. The HBV vaccination coverage in the Afar, Somali and Gambelia regions was found to be below the average in the country. These regions are predominantly inhabited by pastoralist communities, which face higher levels of poverty and have more limited access to healthcare services compared with other parts of the country.^22^ The 2017 national serosurvey suggested that Afar is the region with the highest chronic HBV infection prevalence at 28.8%.^23^ This implies that special emphasis should be given to the Afar region while working to improve HBV vaccination uptake across the country.

The overall HBV vaccination coverage in the country falls below the estimated coverage required to reduce prevalence, approximately 90%, and below the African average HBV vaccine coverage of 77%, indicating that significant improvements are required to achieve adequate protection against infection and decrease HBV prevalence.^24,25^ Despite this, the prevalence of HBV among children aged <5 y was found to be 1.48% compared with the overall prevalence of an estimated 2–8%, which may indicate that infant vaccination is having a population-level impact.^26^ Modelled data suggest that the lack of improvement in infant vaccine coverage could mean that a reduction in the prevalence of HBV to <0.1% in those aged <5 y may not be achieved before 2100 in the WHO Africa region, well beyond the WHO target of 2030.^25^

Our analysis of the 2016 EDHS data demonstrated that older maternal age, improved maternal education, higher wealth index, distance to a healthcare facility not being a problem and complete utilisation of maternal continuum of care were associated with increased odds of complete HBV vaccination in children, while the number of living children in the household was associated with a lower likelihood of receiving HBV vaccination. These results align with research conducted in Nigeria, another highly populous country in sub-Saharan Africa, where receipt of a birth dose of the HBV vaccine is positively associated with maternal education and household wealth.^27^

We found that higher parental education was associated with complete HBV vaccination, emulating associations seen previously, with children who had better overall health having mothers with better education.^24,28^ This could be due to greater utilisation of preventative healthcare, such as prenatal visits, improved knowledge of the benefits of healthcare interventions and an improved ability to communicate with healthcare providers.^24^ The association with maternal education was stronger than paternal education, which may be an indication of the role of mothers in newborn care, with mothers and female members of the family taking the primary carer role for infants and newborns.^29^

Household wealth index was positively and independently associated with HBV vaccination uptake. These results align with existing evidence that children from higher socioeconomic backgrounds are more likely to receive vaccination.^15^ Despite adjusting for education, the wealth index improves vaccination odds. This could indicate that the lack of knowledge surrounding vaccination may not be the primary cause of low coverage in the poorest group. Other causes for this relationship could be the cost of travel to healthcare facilities for vaccination, where mothers with a higher wealth index can afford this travel and those with a lower wealth index cannot. In addition to travel costs, the opportunity costs of the time required to visit healthcare facilities for a three-dose vaccine programme may also be a significant barrier to mothers who cannot afford to take time off work to vaccinate their children. These results support the requirement for vaccine campaigns to target the poorest wealth groups by reducing the costs surrounding vaccine delivery to improve coverage.

Mothers who utilised a complete maternal continuum of care, defined as mothers who accessed ≥4 antenatal care visits, gave birth in a healthcare facility and attended at least one postnatal check and who were closer to a healthcare facility, had increased odds of complete HBV vaccination. Utilisation of healthcare during pregnancy increases exposure to nurses, doctors and other medical professionals during pregnancies and postpartum, allowing more provision of information on infant vaccination. Mothers who lived close to healthcare facilities experienced both reduced travelling times and costs accessing these facilities, thus increasing their utilisation of healthcare services. Our findings suggest that improving access to, as well as the use of healthcare, improves the odds of HBV vaccination, and targeting areas where there are fewer facilities for a wide geographic range could be key to improving vaccine coverage. Utilisation of community healthcare workers has been shown to support equity in immunisation in rural and under-reached communities, and this is a potential pathway to improve coverage in Ethiopia.^30^ In Ethiopia health posts are the first tier of the four-tier healthcare system, and provide a basic primary healthcare contact point. Health posts have implemented the Health Extension Programme, a community-centred approach, since 2003.^5^ However, training facilities and training for Health Extension Workers (HEWs) require improvements to ensure that HEWs are effective in the delivery of vaccinations, healthcare and education.^31^ Additionally, the utilisation of HEWs in more rural areas, to provide education for mothers as well as HBV vaccination delivery, is essential for increasing coverage.^5,31^

Conversely, our results show that an increase in the number of children is negatively associated with the odds of complete vaccination. Previous studies align with these results, where the probability of vaccination decreased with increasing birth order.^32^ This effect could be a consequence of the time required to take each child for a three-dose vaccine, as observed in a small study in rural southern Ethiopia, in which mothers reported that they were too busy caring for other children as a reason why they defaulted on vaccination for their child.^33^ Alternatively, mothers who have larger families may not see HBV vaccination as necessary if their other children did not receive the vaccine and experienced no negative health consequences.

Mass media communication is important for health messaging and is a popular way to deliver public health information in Ethiopia.^34^ Information is usually delivered through television, radio and written materials such as newspapers. Univariable model results align with previous evidence, indicating that increased access to health information allows families to make better informed health decisions during pregnancy, delivery and after birth.^34^ However, this association was attenuated in the multivariable model, potentially due to co-linear effects of education and wealth index on mass media use.

### Strength and limitations

The key strength of this study is that the analyses were performed on a large, nationally representative sample, indicating that our results could be used to inform public health and vaccination campaigns aiming to reduce the incidence and burden of HBV in Ethiopia.

Despite this, this study faced some limitations. The data were recorded in 2016, before the COVID-19 pandemic and the Tigray civil war, and therefore understanding and exposure to vaccination information may have changed since the data were recorded. Up-to-date data are needed for better estimates. Another potential limitation of this study is that although the DHS target population is nationally representative, children with parents belonging to high-risk groups (e.g. people who inject drugs, people who are homeless, and prisoners) that may be less likely to engage with HBV vaccination services may not have been included. Another limitation is that the results related to paternal sociodemographic factors such as paternal education may be affected by missing data (6.7%) and recall bias. Women who have data collected may not know, or remember, the education level that their partner has achieved, which may bias the effect estimate for the odds of complete HBV vaccination among children with varying paternal education. However, the sensitivity analysis showed that removing paternal education from the model did not change the effect estimates of other sociodemographic or obstetric factors on the odds of complete HBV vaccination (Supplementary Table S2). In addition, the effect of maternal education on child HBV vaccination shows similar results to paternal education and therefore is likely to be a good estimation of the effect of parental education on child HBV vaccination status.

## Conclusions and implications

HBV vaccination coverage among infants aged 12–36 mo in Ethiopia is low despite HBV infection being highly endemic in the country. While vaccination for HBV is the primary transmission-prevention route, low infant coverage means additional intervention methods such as screening, disease monitoring and treatment of chronic cases are required to prevent disease spread.

To improve coverage, the identification and targeting of variables that are positively associated with HBV vaccination levels could be important for future public health messaging and vaccine campaigns. Initiatives such as improving accessibility to infant HBV vaccination and participation in formal education, targeting mothers in the poorest wealth groups and women with larger family sizes in vaccination programmes, providing easier access to healthcare facilities for those who live further away, as well as encouraging the use of the maternal continuum of care, could be important strategies that can improve HBV vaccine coverage, leading to a reduction in the future incidence of HBV and alleviating disease burden.

## Supplementary Material

ihaf142_Supplemental_File

## Data Availability

The data that support the findings of this study are not publicly available. Analysis is based on data obtained from the Demographic and Health Survey Program (DHS), which requires registration and formal request for approval. Data may be requested from the DHS Program (https://dhsprogram.com/Data/) in accordance with their data access policies.
