# Supplementary material for: Coverage and determinants of hepatitis B virus vaccine uptake among children aged 12–36 months in Ethiopia: an analysis of the 2016 Ethiopian Demographic and Health Survey
Source: Int Health. 2025 Dec 13;18(5):758–66. doi: 10.1093/inthealth/ihaf142 (PMC13530290; doi:10.1093/inthealth/ihaf142)
Supplement: Supplementary file 1 [file ihaf142_supplemental_file.docx]

**Supplementary Materials**

**Magnitudes and determinants of hepatitis B virus vaccine uptake among children aged 12-36 months in Ethiopia: An analysis of 2016 Ethiopian Demographic and Health Survey**

**Supplementary Methods**

**Sampling for the DHS**

Enumeration Areas (EA) were created for the 2007 Ethiopian Population and Housing census, each containing 181 households (12). The EAs were stratified into urban and rural areas, resulting in 21 sampling strata for the EDHS. Urban EAs with more households were segmented with one segment used for sampling. A two-stage stratified cluster sampling technique was deployed to select households for participation in the study (5). At the first stage, 645 EAs were selected using probability sampling proportional to EA size, with independent selection in each stratum. At the second stage 28 households per cluster were selected for data collection, with equal probability of selection (5).

**Sample**

The sample for this study was taken from the original 10,641 individuals with children under 5-years of age at the time of the DHS study. Of these 10,641 individuals, those whose child had died before the time of the survey (b5), those whose children were not between the ages of 12 and 36 months (b19) and those who did not have data collected on HBV vaccination doses received (h61, h62, h63) were excluded. This left a sample of 3,855 individuals who were used in the analysis for this study.

**Weighting variable**

The sample weight variable (v005) created by the DHS for individual women sample weight was used to create this study’s sample weight variable for analysis. This was transformed by dividing v005 by 1000000 (5).

**Exposure variables**

The exposure variables used in this study were maternal age at birth, calculated using the date of birth of the child (b3) and the date of birth of the mother (v011) to record maternal age at birth of child in years.

The maternal education exposure variable used was the highest educational attainment variable (v106) which was coded in the original dataset into no education, primary education, secondary education and higher.

The paternal education exposure variable used was the highest educational attainment variable (v701) coded into no education, primary, secondary, higher and don’t know. Questions were answered by the women residing the household where some were unaware of the paternal educational attainment level. Those who answered “don’t know” were recoded to missing values.

The DHS wealth index variable (v190) is a composite measure of a households cumulative living standard. This was categorised into poorest, poorer, middle, richer and richest and this variable was used for this study analysis.

The marital status variable (v501) was taken from the original dataset. The variable was originally coded into six categories but compressed into three; “never in union”, “married or living together” and “previously married or separated” for this analysis.

The mass media exposure was a created from a combination of three variables (v157, v158, v159) which recorded data on frequency of access to newspaper and magazines, radio and tv. The original variables categorised use of these media into “not at all”, “less than once a week”, “once a week” and “almost every day”. Data show that there was none in the sample who accessed any of these media more than once a week, therefore the mass media variable was dichotomised into a yes/no answer for those who access mass media and those who do not. Those who answered once a week to either newspaper, radio or tv were categorised as accessing mass media and those who answered less than once a week or not at all were categorised as not accessing mass media.

The distance to healthcare facility variable (v467d) was extracted from the original DHS file and originally coded into “no problem”, “big problem” and “not a big problem”. All respondents either answered with “big problem” or “not a big problem”, which indicated they were either further from healthcare facility or closer to a healthcare facility respectively.

The maternal continuum of care variable was a combination of the antenatal care visits (m14), location of delivery (m15) and postnatal care visits (m70). Using Alamneh et al., 2022 study, the maternal continuum of care variable was dichotomised, with those who accessed maternal continuum of care had 4 or more antenatal care visits, gave birth in a healthcare facility and had a postnatal check done on the baby. For mothers who missed any of these three services they were considered not to have accessed the maternal continuum of care.

Finally, the variable number of living children (v218) was extracted from the original DHS dataset and used in this analysis.

**Outcome variable**

The DHS variables used to create the final outcome variable for HBV vaccine status were records for each HBV vaccine dose received (h61, h62, h63), for dose 1, 2 and 3 respectively. The original HBV dose received variables were coded into “no”, “vaccination date on card”, “reported by mother”, “vaccination marked on card”, “don’t know”.

As per the Guide to DHS statistics (DHS-7, version 2) provided by the demographic and health surveys program: where information is present on the card, this is taken as the dose received, if there is no vaccination card then the mothers report of vaccination is taken as dose received. A vaccination is considered given if information is provided by either source. Therefore, these variables were recoded into “No vaccination”, “Vaccination” and “Don’t know” for each dose of the HBV vaccine. For this study, those who responded “don’t know” were considered not have received that does of the vaccine.

The final outcome variable, HBV vaccination status, was coded into “No vaccination” for children who did not receive a single dose of the HBV vaccine, “Incomplete vaccination” for children who had received one or two doses of the HBV vaccine and “Complete vaccination” for children who received all three doses of the HBV vaccine. The response variable was classified into ordered categories therefore, ordinal logistic regression was selected.

**All variables extracted from original EDHS dataset**

| Original variable name | Original variable label |
| --- | --- |
| b19 | Current age of child in months |
| b5 | Child is alive |
| v005 | Women’s individual sample weight |
| b3 | Date of birth (cmc) - child |
| v011 | Date of birth (cmc) - mother |
| v106 | Highest education level - mother |
| v701 | Highest education level - father |
| v190 | Wealth index combined |
| v501 | Current marital status |
| v157 | Frequency of reading newspaper or magazine |
| v158 | Frequency of listening to radio |
| v159 | Frequency of watching television |
| v467d | Getting medical help for self: Distance to health facility |
| m14 | Number of antenatal visits during pregnancy |
| m15 | Place of delivery |
| m70 | Baby postnatal check within 2 months |
| v218 | Number of living children |
| h61 | Received hepatitis b1 |
| h62 | Received hepatitis b2 |
| h63 | Received hepatitis b3 |

**Statistical analysis – Model assumptions**

The Brant test for the proportional odds assumption showed that the odds ratio held constant across the cut-off points of HBV vaccination status at 5% significance level for each univariable model and the final multivariable model (p-value = 0.261). This indicates that the odds ratio obtained by modelling complete vaccination versus no vaccination/incomplete vaccination; and incomplete/complete vaccination versus no vaccination were not dissimilar

**Other characteristics of HBV vaccine uptake**

Approximately 41.8% of children whose fathers had no formal education had not received any dose of HBV vaccine compared to 22.4% of children whose fathers received at least primary education. Roughly half (50.2%) of children from households in the poorest wealth quantile had no HBV vaccine dose compared to 9.7-27.0% of children in the poorer to richest wealth indices. Conversely, 82.8% and 70.0% of children whose mothers and fathers, respectively, had received higher education had complete vaccination against HBV. Only half (49.9%) of children from mothers with incomplete maternal continuum of care utilisation were fully vaccinated against HBV compared to 83.2% of children from mothers who had complete utilisation (Table 1).

**Univariable Results**

Univariable analysis showed maternal age at birth, maternal education, paternal education, wealth index, exposure to mass media, distance to healthcare facility, maternal continuum of care and number of living children were all factors which influence odds of HBV vaccination and were included in a multivariable analysis. In the multivariable model, maternal age at the birth, maternal education (primary, secondary), paternal education (primary), wealth index, distance to a healthcare facility and maternal continuum of care were all associated with an increased odds of HBV vaccination. An increase in the number of living children was associated with a decreased odds of HBV vaccination.

**Interpretation of spatial analysis results**

The geographic distribution of complete HBV vaccination in Ethiopia from the 2016 EDHS is shown in main text Figure 1. We might be able to locate clusters that have a higher or lower rate of complete HBV vaccination from this map, but it is subjective from individual to individual. In contrast, the Getis-ord Gi* statistics (main text Figure 2) objectively identify the hot spot and cold spot clusters at different levels of alpha (1%, 5% or 10%), with the term hot spot used to denote higher rates of no or incomplete HBV vaccination. This method provides a more precise classification of areas as hot spots or cold spots.

**Supplementary Figures**

**Supplementary Figure S1.** Global spatial autocorrelation of complete HBV vaccination among children in Ethiopia, produced using Arc GIS version 10.7.

**Supplementary Tables**

**Supplementary Table S1:** Distribution of parental sociodemographic and obstetric factors for children aged 12-36 months in Ethiopia.

| Variable | Category | Frequency | Proportion (%) |
| --- | --- | --- | --- |
| Sex of child | Male | 1957 | 50.77 |
|  | Female | 1898 | 49.23 |
| Maternal age at birth of child | 12-15 | 33 | 0.86 |
|  | 16-20 | 685 | 17.77 |
|  | 21-25 | 1077 | 27.94 |
|  | 26-30 | 1009 | 26.17 |
|  | 31-35 | 615 | 15.95 |
|  | 36-40 | 336 | 8.72 |
|  | 41-45 | 91 | 2.36 |
|  | 46-50 | 9 | 0.23 |
| Maternal education | No education | 2379 | 61.71 |
|  | Primary | 1019 | 26.43 |
|  | Secondary | 294 | 7.63 |
|  | Higher | 163 | 4.23 |
| Paternal education | No education | 1730 | 47.68 |
|  | Primary | 1173 | 32.33 |
|  | Secondary | 399 | 11.00 |
|  | Higher | 300 | 8.27 |
|  | Don’t know | 26 | 0.72 |
| Wealth index | Poorest | 1435 | 37.22 |
|  | Poorer | 640 | 16.60 |
|  | Middle | 524 | 13.59 |
|  | Richer | 463 | 12.01 |
|  | Richest | 793 | 20.57 |
| Marital status | Never in union | 29 | 0.75 |
|  | Married/living together | 3628 | 94.11 |
|  | Previously married/separated | 198 | 5.14 |
| Mass media exposure | No | 3038 | 78.81 |
|  | Yes | 817 | 21.19 |
| Distance to health facility | Not a big problem | 1780 | 46.17 |
|  | A big problem | 2075 | 53.83 |
| Maternal continuum of care | No | 3706 | 96.13 |
|  | Yes | 149 | 3.87 |
| Number of living children | 1 | 746 | 19.35 |
|  | 2 | 764 | 19.82 |
|  | 3 | 590 | 15.30 |
|  | 4 | 528 | 13.70 |
|  | 5 | 415 | 10.77 |
|  | 6 | 324 | 8.40 |
|  | 7 | 241 | 6.25 |
|  | 8 | 132 | 3.42 |
|  | 9 | 74 | 1.92 |
|  | 10 | 28 | 0.73 |
|  | 11 | 11 | 0.29 |
|  | 12 | 2 | 0.05 |

**Supplementary Table S2:** Adjusted Odds ratio for sensitivity analyses, ordinal logistic regression testing association between sociodemographic and obstetric factors and HBV vaccination status of children (12-26 months) in Ethiopia, without paternal education.

| **Variable** | **Category** | **AOR (95% CI)** | **P-value (AOR)** |
| --- | --- | --- | --- |
| **Maternal age** |  | 1.05 (1.03, 1.07) | <0.001 |
| **Maternal education** | No education | 1 |  |
|  | Primary | 1.74 (1.39, 2.19) | <0.001 |
|  | Secondary | 2.51 (1.42, 4.43) | <0.001 |
|  | Higher | 2.07 (0.87, 4.96) | 0.10 |
| **Wealth index** | Poorest | 1 |  |
|  | Poorer | 1.55 (1.18, 2.02) | <0.001 |
|  | Middle | 1.53 (1.16, 2.02) | <0.001 |
|  | Richer | 1.76 (1.32, 2.35) | <0.001 |
|  | Richest | 2.44 (1.62, 3.68) | <0.001 |
| **Marital status** | Never in union | 1 |  |
|  | Married/Living with partner | 1.35 (0.50, 3.62) | 0.55 |
|  | Previously married | 0.85 (0.29, 2.47) | 0.77 |
| **Mass media exposure** | No | 1 |  |
|  | Yes | 1.28 (0.93, 1.78) | 0.13 |
| **Distance to health facility** | Big Problem (Further) | 1 |  |
|  | Not a big problem (Closer) | 1.54 (1.26, 1.88) | <0.001 |
| **Maternal continuum of care** | No | 1 |  |
|  | Yes | 2.87 (1.47, 5.62) | <0.001 |
| **Number of living children** |  | 0.85 (0.79, 0.92) |  |
| **Cut 1**  **Cut 2** |  | 2.43 (0.82, 7.16)  6.60 (2.23, 19.50) | 0.11  <0.001 |
